# Correction: Removal of fluoride ions using a polypyrrole magnetic nanocomposite influenced by a rotating magnetic field

**DOI:** 10.1039/d0ra90010a

**Published:** 2020-01-23

**Authors:** Uyiosa Osagie Aigbe, Robert Birundu Onyancha, Kingsley Eghonghon Ukhurebor, Kingsley Onyebuchi Obodo

**Affiliations:** Department of Physics, College of Science, Engineering and Technology, University of South Africa Pretoria South Africa; School of Physical Sciences and Technology, Technical University of Kenya Kenya 08muma@gmail.com +254722545854; Climatic/Environmental/Telecommunication Unit, Department of Physics, Edo University Iyamho Edo Nigeria; HySA Infrastructure Centre of Competence, Faculty of Engineering, North-West University South Africa

## Abstract

Correction for ‘Removal of fluoride ions using a polypyrrole magnetic nanocomposite influenced by a rotating magnetic field’ by Uyiosa Osagie Aigbe *et al.*, *RSC Adv.*, 2020, **10**, 595–609.

The authors regret that Table 1 was displayed incorrectly in the original article, as the values for the Temkin constants were not correctly aligned with their respective columns. The corrected version of Table 1 is shown below.

**Table 1:** Freundlich and Temkin constants for fluoride ions adsorption

Freundlich isotherm			Temkin isotherm		
*K*_L_ (mg g^−1^)	*n*	*R* ^2^	*K*_T_ (L mg^−1^)	*b*_T_ (kJ mol^−1^)	*R* ^2^
1.11740	1.34171	0.98381	1.0153	22.5	0.97579

The Royal Society of Chemistry apologises for these errors and any consequent inconvenience to authors and readers.

## Supplementary Material

